# Speckle tracking echocardiography-determined measures of global and regional left ventricular function correlate with functional capacity in patients with and without preserved ejection fraction

**DOI:** 10.1186/1476-7120-11-20

**Published:** 2013-06-14

**Authors:** John W Petersen, Talha F Nazir, Licheng Lee, Cynthia S Garvan, Ashkan Karimi

**Affiliations:** 1 Division of Cardiovascular Medicine, University of Florida, Gainesville, Florida, USA; 2 Health Science Center, University of Florida, Gainesville, Florida, USA

**Keywords:** Left ventricular function, Speckle tracking echocardiography, Strain, Heart failure

## Abstract

**Background:**

Standard measures of left ventricular systolic and diastolic function often fail to identify left ventricular dysfunction in patients with heart failure and do not correlate with measures of functional capacity.

**Aim:**

To determine if speckle tracking echocardiography (STE)–determined measures of global and regional myocardial contractility have a linear association with functional capacity in patients with and without preserved ejection fraction.

**Methods:**

In 68 adult patients, functional status was estimated with the Duke Activity Status Index (DASI), left ventricular ejection fraction was determined with Simpson’s biplane method, and QLAB advanced quantification software (Philips, The Netherlands) was used to determine peak measures of strain.

**Results:**

Global and regional measures of longitudinal, circumferential, and radial strain had a strong linear association with the DASI score. Longitudinal strain in the inferolateral segments had the strongest correlation with DASI (r = −0.72, P < 0.001). In patients with an ejection fraction ≥45%, ejection fraction and E/e’ had no correlation with DASI, whereas longitudinal strain in the inferolateral segments had significant correlation with DASI (r = −0.53, P = 0.03, n = 16).

**Conclusions:**

STE–determined measures of global and regional left ventricular function have a strong linear association with estimates of functional capacity in patients with and without preserved ejection fraction. STE–determined measures of strain, especially longitudinal strain, are likely to be important targets for therapy and should be considered in future studies aimed at improving our diagnosis of left ventricular inadequacy in patients with heart failure, especially those with preserved ejection fraction.

## Background

Measurements of left ventricular (LV) function are important for estimating prognosis and guiding the management of patients with multiple forms of cardiac disease [1]. LV systolic function is most commonly reported as an ejection fraction (EF). However, EF at times fails to document the LV dysfunction responsible for heart failure symptoms [2]. Further, previous work has shown that EF does not correlate with quantitative measures of functional capacity [3,4]. In patients with heart failure and preserved EF, researchers have suggested that abnormal Doppler echocardiographic measures of LV diastolic filling and myocardial relaxation velocities are able to quantify the abnormal myocardial compliance responsible for symptoms. However, these Doppler echocardiographic measures of LV diastolic function are often normal in patients with heart failure and preserved EF [5].

Advances in non-invasive imaging have allowed for newer measurements of LV function that have enhanced the understanding of the mechanical deficits that may lead to clinically apparent LV dysfunction. These newer measures of myocardial contractility include quantification of the deformation of a myocardial segment using speckle tracking echocardiography (STE) [6]. STE allows determination of the magnitude of deformation of all myocardial segments along all three orthogonal axes of the heart (radial, circumferential, and longitudinal) [7]. In patients with heart failure and preserved EF, STE-determined measures of average myocardial contractility were reduced, suggesting that these newer measures of contractility may be better than EF in quantifying LV dysfunction [8]. Yet, whether these more sophisticated measures of LV function correlate with functional capacity remains unclear. We aimed to determine if global and regional measures of strain had a linear association with a score of functional capacity in patients with and without preserved EF.

## Methods

### Patients

This study was approved by the University of Florida institutional review board. Adult patients who were referred for an echocardiogram for clinically indicated reasons and who had echocardiographic images that were determined to be adequate for evaluation of regional LV systolic function were approached for enrollment. The patients’ functional status was estimated with the Duke Activity Status Index (DASI) questionnaire [9]. DASI is a 12-item questionnaire which asks if a patient can comfortably complete different activities. Patients receive points for every activity that can be completed comfortably, and points received for each activity are weighted based on the associated metabolic equivalent of each activity. Possible scores range from 0 to 58.2 points. These scores correlate with peak oxygen uptake. Subjects were also classified into groups as follows: controls = no symptoms with physical activity and EF >55% and no evidence of diastolic dysfunction; Group A = no symptoms with physical activity but EF ≤55% or diastolic dysfunction; Group B = symptoms with ordinary activity, such as walking more than 2 blocks; Group C = symptoms with less than ordinary activity. Diastolic dysfunction was defined in accordance with the American Society of Echocardiography recommendations [10]. Patients with moderate or severe valve disease were excluded. Further, those patients with a documented non-cardiac cause of a decreased functional capacity, such as interstitial lung disease, were excluded. In an effort to determine if traditional Doppler echocardiographic parameters of diastolic function correlate better with functional status than do STE measures, a secondary analysis was performed and limited to patients with normal or mildly depressed LV systolic function, defined as an LV EF ≥45%.

### Echocardiography

Echocardiograms were obtained with an iE33 (Philips, The Netherlands). Parasternal short axis views were obtained at the basal, midventricular, and apical levels [11]. Additionally, three standard apical views (4-chamber, 2-chamber, and 3-chamber) and a parasternal long axis image were obtained. To optimize speckle tracking, images were acquired at as high a frame rate as possible (50–90 frames/s). LV end-systolic and end-diastolic volumes and ejection fraction were determined by manual tracing of the end-systolic and end-diastolic endocardial borders using apical 4- and 2-chamber views, employing Simpson’s biplane method. Pulsed wave Doppler at the level of the mitral valve leaflet tips was used to determine peak early (E) and atrial (A) filling velocities, and deceleration time (DT). Tissue Doppler was used to determine peak early (e’) velocity of the medial mitral annulus. Function of each visualized LV segment was qualitatively evaluated, and a wall motion score was assigned for each LV segment as follows: normal function = 1, mild hypokinesis = 1.5, moderate hypokinesis = 2, severe hypokinesis = 2.5, akinesis = 3, dyskinesis = 4. A wall motion score index was calculated as the average wall motion score for all of the visualized LV segments.

### Speckle tracking analysis

Analysis was performed using QLAB advanced quantification software version 7.1 (Philips, The Netherlands). Automated tracking of myocardial speckles was reviewed and manually adjusted as minimally as possible. The tracking quality of each segment was visually evaluated and if tracking was felt to be inaccurate, strain analysis of that segment was not included. Speckle tracking analysis provided measures of peak circumferential, radial, and longitudinal strain in 18 LV segments (6 basal, 6 mid, and 6 apical) and rotation in the basal and apical LV regions. Strain was determined for the entire myocardial segment, not just the endocardial or epicardial portion. LV twist was calculated as the maximal apical rotation minus the maximal basal rotation. Average global circumferential, radial, and longitudinal strain were calculated. Further, average circumferential, radial, and longitudinal strain in each of the 6 walls of the LV was calculated. If fewer than 2/3 of the variables for a computed variable were available, then that computed variable was not included in the analysis. QLAB software also used the automated endocardial borders to determine the fractional area change (FAC) in each of the LV regions (basal, mid, and apical) by comparing the LV luminal area at end-diastole and end-systole.

### Statistical analysis

Analysis was completed with SPSS Version 20. Continuous variables were compared between groups using a Kruskal Wallis test. Continuous variables were compared between groups A and B using a Mann–Whitney U test. Correlations between continuous variables are presented as Pearson correlation coefficients. The magnitude of correlation coefficients between EF/DASI and EF/regional measures of LV strain were then compared [12]. Categorical variables were compared using Chi-Square or Fisher’s Exact test as appropriate. Multiple linear regression was used to determine if the association between strain variables and the DASI score of functional capacity persisted after adjusting for the baseline variables that were different between the groups (age and glomerular filtration rate). A *P* value <0.05 was considered statistically significant.

## Results

### Patients

A total of 88 patients were enrolled in this study. Seven patients with moderate or severe valve disease were excluded from analysis. Further, 13 patients with clear non-cardiac causes of a decreased functional capacity, such as interstitial lung disease, were excluded from the analysis. Of the remaining 68 patients, 19 were identified as controls as they had no symptoms and normal LV systolic and diastolic function as described above. The baseline characteristics for the 19 controls and 49 patients with a reduced functional capacity or asymptomatic LV systolic or diastolic function are shown in Table 1. In the 49 patients, the primary indication for the echocardiogram was a history of cardiomyopathy, shortness of breath, or edema in 55%, chest pain in 18%, syncope in 8% and abnormal rhythm in 6%. In the 35 patients with an LV EF ≤55%, 23% had a clear ischemic mechanism for LV systolic dysfunction, whereas 77% did not have a clearly documented ischemic mechanism.

**Table 1:** Baseline characteristics

	**Total**	**Controls**	**Group A**	**Group B**	**Group C**	
**Variable**	**N = 68**	**N = 19**	**N = 7**	**N = 26**	**N = 16**	P value*
Age (years)	42.7 (16)	30.1 (12.7)	36.9 (19)	47 (13)	53 (14)	<0.001
Height (inches)	67 (4.3)	67 (3.4)	70 (4.8)	67 (4.3)	68 (4.7)	0.40
Weight (lbs)	167 (33)	151 (28)	158 (34)	170 (29)	182 (39)	0.10
SBP (mmHg)	123 (21)	120 (22)	122 (13)	122 (21)	127 (24)	0.54
DBP (mmHg)	76 (14)	74 (13.7)	75 (10.4)	74 (15)	82 (14)	0.22
Cr (mg/dL)	1.5 (2.2)	0.78 (0.2)	2.9 (4.3)	1.0 (0.5)	2.3 (3.3)	0.03
GFR (mL/min)	90 (41)	115 (35)	70 (41)	94 (37)	69 (39)	0.01
Male	60%	53%	71%	58%	69%	0.74
Caucasian	44%	50%	75%	36%	40%	0.23
Tobacco use	38%	21%	0%	50%	56%	0.02
Diabetic	12%	0%	0%	15%	25%	0.09
Hyperlipidemia	22%	5%	14%	19%	50%	0.02
DASI	38.6 (20)	58.2 (0)	58.2 (0)	37 (9.2)	9.9 (6)	<0.001
EF (%)	51.4 (14)	60.4 (3)	51.7 (3)	52 (12)	39.8 (19)	<0.001
e’	8.5 (3)	10.4 (2.8)	10.8 (2.6)	7.7 (2.3)	6.5 (2.7)	0.001
E/e’	13 (8.8)	9.7 (3.1)	8.4 (2.5)	12 (4.6)	20 (14.6)	0.004
GLS	–15 (4.8)	–18.0 (2.4)	–17.5 (1.8)	–14.5 (3.8)	–10.3 (6.7)	0.016
GCS	–17.7 (5.5)	–21.4 (2.4)	–19.2 (1.3)	–17.9 (4.7)	–13.4 (6.8)	0.003
GRS	21.1 (13)	30.0 (7.5)	35.0 (15)	20.7 (11.5)	10.7 (7.9)	<0.001

Continuous variables are presented as mean (standard deviation).

Abbreviations: *Cr* creatinine, *DBP* diastolic blood pressure, *E* peak early left ventricular filling velocity, *e'* peak early mitral annular velocity, *GFR* glomerular filtration rate, *SBP* systolic blood pressure.

*P value comparing all groups.

### Speckle tracking echocardiography strain analysis feasibility and reliability

Speckle tracking echocardiography strain analysis was attempted on 1224 LV segments (18 segments for each of 68 patients). Importantly, because of inadequate tracking quality, STE-derived measures of LV function could not be determined for all segments. Circumferential strain could be determined in 913 (75%), radial strain in 781 (64%), and longitudinal strain in 804 (66%) of the LV segments analyzed. To test the inter-observer reliability between the two investigators who completed strain analysis for this project (JP,TN), each independently determined circumferential, radial, and longitudinal strain in a total of 48 LV segments from 8 different subjects. Inter-observer reliability was very strong for longitudinal strain (r = 0.91, average difference 0.5 ± 3%) and good for circumferential strain (r = 0.8, average difference 0.65% ± 4.8%) and was similar to previously reported inter-observer reliability of speckle software [13]. In our study, inter-observer reliability was weakest for radial strain (r = 0.85, average difference 0.17% ± 10%).

### Correlation of echo measures of global LV function with functional status

In the 49 patients with a reduced functional capacity or asymptomatic LV systolic or diastolic function (Groups A, B, and C), global longitudinal, radial, and circumferential strain had a strong linear association with the DASI score of functional status (Table 2). The linear association between these global measures of strain and the DASI score of functional status remained significant after controlling for age and glomerular filtration rate in a multiple linear regression model, whereas age and GFR did not provide prediction of DASI score independent of the effects of global measures of strain. However, the strength of the correlations between these global strain measures and DASI score was not significantly different than the strength of the correlation of EF with DASI score in this small cohort (Table 2). The strength of the correlations between automated measures of FAC and the DASI score was not significantly different than the strength of the correlation of manually determined biplane EF with DASI score (basal FAC: r = 0.44, n = 44, P = 0.003; mid FAC: r = 0.46, n = 49, P = 0.001; apical FAC: r = 0.44, n = 49, P = 0.002). Similarly, the strength of the correlation of the wall motion score index, determined by qualitative assessment of myocardial contractility in all visualized LV segments, with the DASI score (r = −0.5, P < 0.001) was not different than the strength of the correlation between other global measures of LV function and DASI score. LV twist had a weak correlation with DASI score.

**Table 2:** Correlation of speckle tracking echocardiography (STE) measures of global left ventricular function with DASI score of functional status

**STE variable**	**Correlation of STE variable with DASI**	**P value**	**N**	**Correlation of EF with DASI in same subjects**	**P value**
Global Circ. Strain	–0.48	0.002	39	0.51	0.001
Global Rad. Strain	0.68	<0.001	32	0.58	0.001
Global Long. Strain	–0.57	0.002	26	0.51	0.008
LV Twist	0.33	0.07	31	0.66	<0.001

Abbreviations: *Circ.* circumferential; *DASI* Duke Activity Status Index, *EF* ejection fraction, *Long.* longitudinal, *LV* left ventricular, *Rad*. radial, *STE* speckle tracking echocardiography.

### Correlation of regional STE measures with functional status

**Figure 1:**
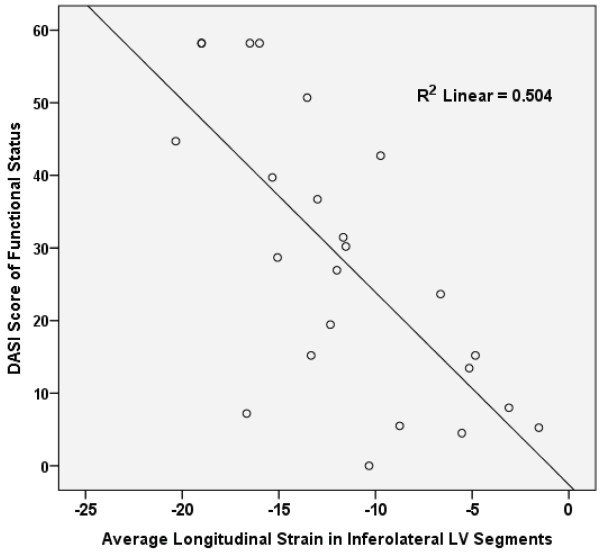
**Correlation of longitudinal strain in the inferolateral segments with functional status.** Scatter plot demonstrating correlation of longitudinal strain in the inferolateral segments and the Duke Activity Status Index (DASI) score of functional status.

In the patients included in this study, longitudinal, circumferential, and radial strain in the various walls of the LV had significant linear associations with the DASI score of functional status (Table 3). As detailed in Table 3, the linear association between most of these regional measures of strain and the DASI score of functional capacity remained significant after controlling for age and glomerular filtration rate in a multiple linear regression model, whereas age and GFR did not provide prediction of DASI score independent of the effects of regional measures of strain. In nearly all of the walls of the LV, longitudinal strain provided the strongest correlation with functional status. Longitudinal strain in the inferolateral segments had the strongest correlation with the DASI score of functional capacity (r = −0.72, P < 0.001) (Figure 1). The strength of the correlations between most of these regional measures of LV strain and DASI score was not found to be significantly different than the strength of the correlation of EF with DASI score (Table 3). However, the strength of the correlation between anteroseptal longitudinal strain and the DASI score was stronger than the correlation of EF with DASI score in the same subjects, with borderline statistical significance (r = 0.66 vs. r = 0.43, n = 34, P = 0.052).

**Table 3:** Correlation of speckle tracking echocardiography (STE) measures in each wall of the left ventricle and DASI score of functional status

**STE variable**	**Correlation of STE variable with DASI**	**P value**	**N**	**Correlation of EF with DASI in same subjects**	**P value**
Long. strain AS^*†‡^	–0.64	<0.001	34	0.43	0.011
Long. strain Ant^*^	–0.66	0.003	18	0.59	0.01
Long. strain AL	–0.36	0.097	22	0.40	0.07
Long. strain IL^*†‡^	–0.71	<0.001	24	0.59	0.002
Long. strain Inf^*†^	–0.53	<0.001	43	0.47	0.002
Long. strain IS^*^	–0.43	0.003	46	0.40	0.006
Circ. strain AS^*†^	–0.46	0.001	47	0.46	0.001
Circ. strain Ant^†^	–0.40	0.03	30	0.53	0.003
Circ. strain AL	–0.39	0.032	31	0.54	0.002
Circ. strain IL	–0.12	0.44	46	0.46	0.001
Circ. strain Inf^*^	–0.44	0.003	45	0.51	<0.001
Circ. strain IS^*†^	–0.58	<0.001	44	0.48	0.001
Rad. strain AS^*†^	0.46	0.001	45	0.46	0.001
Rad. strain Ant	0.44	0.05	20	0.46	0.04
Rad. strain AL^†^	0.52	0.008	25	0.50	0.012
Rad. strain IL^*^	0.41	0.007	42	0.48	0.001
Rad. strain Inf^*†‡^	0.67	<0.001	36	0.56	<0.001
Rad. strain IS^*†^	0.49	0.002	38	0.56	<0.001

Abbreviations: *AS* anteroseptal, *Ant* anterior, *AL* anterolateral, *Circ.* circumferential, *DASI* Duke Activity Status Index, *EF* ejection fraction, *Inf* inferior, *IL* inferolateral, *IS* inferoseptal, *Long.* longitudinal, *Rad.* radial, *STE* speckle tracking echocardiography.

^*^Linear association between regional measure of strain and DASI score remained significant after controlling for age and glomerular filtration rate in a multiple linear regression model.

^†^P value <0.05 when comparing regional measure of strain between groups A, B and C.

^‡^P value <0.05 when comparing regional measure of strain between group A and B.

**Figure 2:**
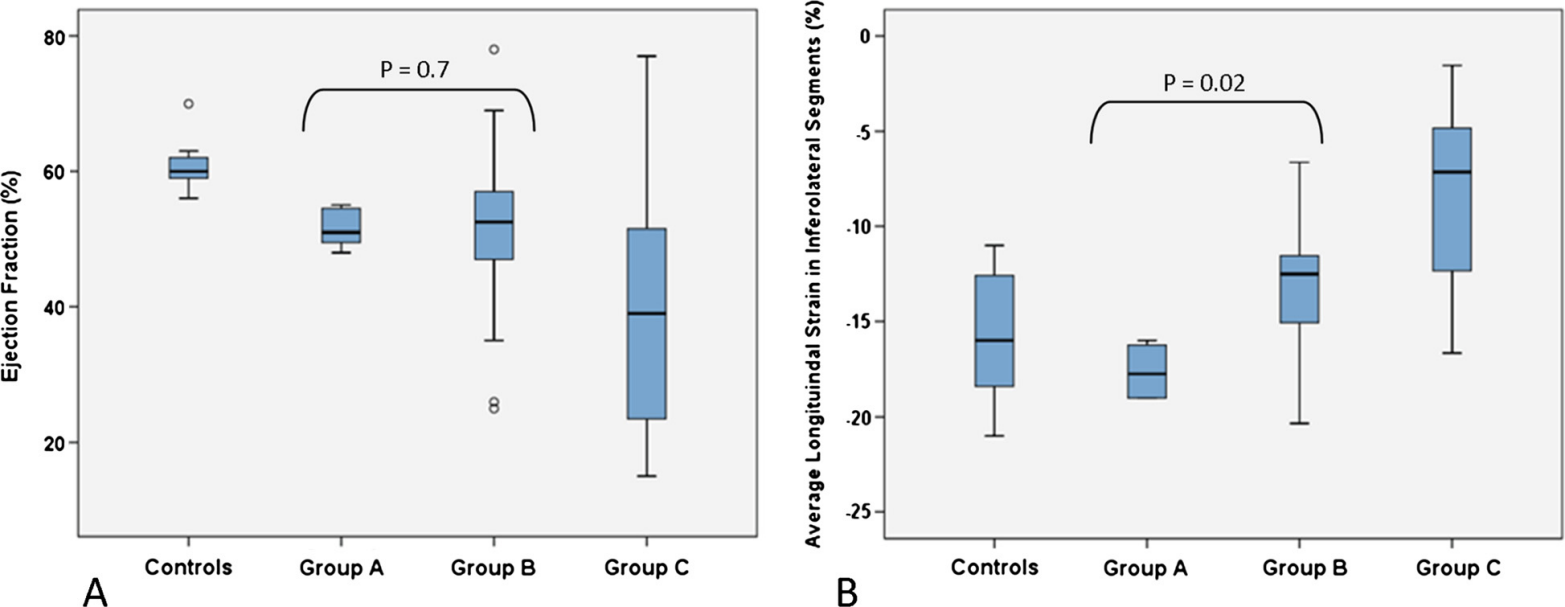
**Ejection fraction and average longitudinal strain in inferolateral left ventricular segments for each patient group.** Box plots demonstrating the median (horizontal line), interquartile range (box) and range (whiskers) of **A**. ejection fraction, and **B**. average longitudinal strain in inferolateral left ventricular segments for controls, patients with abnormal left ventricular ejection fraction or diastolic function but no symptoms (Group A), patients with symptoms with ordinary activity (Group B), and patients with symptoms with less than ordinary activity (Group C).

As shown in Table 3, nearly all STE measures in the various walls of the LV were significantly different between those patients in groups A, B, and C. Further, some regional measures of longitudinal and radial strain were different between those patients without symptoms (group A) and those patients with mild symptoms (group B), whereas EF was not different between these groups (Figure 2). Only one patient without symptoms (group A) had qualitatively determined wall motion abnormalities (global mild hypokinesis). The predominant qualitatively determined wall motion abnormality in those patients with symptoms (groups B, C) was in the anteroseptal, inferolateral, inferior, or inferoseptal walls of the LV. The pattern of qualitatively determined wall motion abnormalities was similar between those patients with mild (group B) and moderate (group C) symptoms.

### Correlation of STE measures with functional status in patients with an EF ≥45%

In the 34 patients included in this study who had an EF ≥45%, biplane EF did not have a linear association with the DASI score of functional status (r = 0.064, P = 0.72, n = 34). Further, traditional echocardiographic measures of “diastolic function”, such as E/e’, had no correlation with the DASI score of functional status (r = −0.16, P = 0.37, n = 32). However, similar to what was shown in all patients, longitudinal strain in the inferolateral segments had significant correlation with DASI score in these patients with preserved EF (r = −0.53, P = 0.03, n = 16).

## Discussion

We aimed to determine if novel measures of LV contractility had a strong linear association with functional capacity. STE-determined measures of global and regional LV function had a strong linear association with the DASI score of functional capacity. The strength of the association between most STE-determined measures of strain and the DASI score of functional capacity was not statistically different than the association of EF with the DASI score in this small cohort. However, the difference in the strength of the correlation of anteroseptal longitudinal strain with the DASI score and the strength of the correlation of EF with the DASI score was near statistically significant despite the small sample. Further, in patients with preserved EF, STE-determined measures of regional strain had a strong correlation with functional capacity, whereas EF and traditional Doppler echocardiographic parameters of LV diastolic function had no correlation with functional capacity. These findings support previous work which has demonstrated decreased systolic myocardial strain in patients with heart failure but preserved EF [8,14]. Finally, multiple measures of STE-determined global and regional strain were different between those patients with no symptoms (Group A) and those patients with mild symptoms (Group B). These findings suggest that STE measures of global LV myocardial contractility may have better sensitivity in detecting clinically relevant LV dysfunction, especially in those patients with heart failure but preserved EF.

Regional longitudinal strain appeared to have stronger correlation with functional capacity than did radial or circumferential strain both in the entire cohort and in just those with preserved EF. Previous work has shown that longitudinal motion of the LV is an important aspect of normal and efficient LV contraction [15]. While there are many methods available to measure longitudinal motion of the LV, longitudinal strain appears to be superior to mitral annular displacement in detecting subtle deficits in LV contraction after myocardial injury [16].

The strong correlation of longitudinal strain in the inferolateral and anterior/anteroseptal walls of the LV with the DASI score of functional status could be explained by an important aspect of efficient blood flow through the LV. In normal hearts the inflow of blood into the LV gives rise to rotational/vortical blood flow patterns. The dominant vortex is under the anterior mitral leaflet, and this vortex gives rise to asymmetric flow of blood in the LV during diastole with redirection of blood toward the superior and medially located LV outflow tract. This flow pattern allows for efficient circulation by preventing collision of flow, and by allowing the slinging of blood toward the LV outflow tract just prior to ejection [17]. Effective longitudinal lengthening of the inferolateral segments during diastole may be required to accelerate the blood coming into the LV, through the inferolaterally located mitral valve, to speeds required for vortex formation. In abnormal hearts the anterior vortex is shorter and more circular with blood being directed away from the LV outflow tract at end diastole [18]. Longitudinal shortening during systole in the anterior and anteroseptal myocardial segments, near the LV outflow tract, may be required to compensate for inefficient flow patterns in which blood is not already directed toward the LV outflow tract. Future studies aimed at determining the relationship of regional strain patterns and intra-LV vortex characteristics are important.

There are limitations to this study. First, our measure of functional status was provided through a questionnaire. Although the DASI score of functional status has been shown to correlate with peak oxygen consumption, has been used as a marker of improved function after various interventions, and recently was shown to have superior prediction of outcome as compared to a 6 minute walk test [19], the questionnaire is not an objective measure of functional status. While we attempted to exclude patients with clear non-cardiac causes of dyspnea and adjusted our analysis according to patient’s age and glomerular filtration rate, it is possible that symptoms of dyspnea reported by the patients in this cohort were related to non-cardiac causes. The findings of this study should be confirmed by correlating STE measures of LV function with objective measures of functional reserve, such as MVO2. Second, the sample size for this study was small and therefore the power to detect a difference in the strength of correlation between novel measures of LV function with the DASI score of functional capacity was limited. It is also important to note that STE was not possible in all segments in many patients. A larger sample size will allow for further restriction of analysis to only those patients who have all STE measures of interest available so that confirmation of which global or regional measures of LV contraction have the strongest correlation with functional status can be completed. Finally, as our study was cross-sectional, future studies are necessary to evaluate the ability of unique patterns of LV contraction to predict future cardiovascular events.

## Conclusions

STE-determined measures of global and regional LV function have a strong linear association with the DASI score of functional capacity in patients with and without preserved EF. In patients with preserved EF, STE-determined measures of regional strain have a strong linear association with functional capacity, whereas EF and traditional Doppler echocardiographic parameters of LV diastolic function have no correlation with functional capacity. STE-determined measures of strain, especially longitudinal strain, are likely to be important targets for therapy and should be considered in future studies aimed at improving our diagnosis of LV inadequacy in patients with heart failure, especially those with preserved EF.

## Abbreviations

DASI: Duke Activity Status Index; E: Peak early left ventricular filling velocity; e': Peak early mitral annular velocity; EF: Ejection fraction; LV: Left ventricular; STE: Speckle tracking echocardiography.

## Competing interests

The authors declare no competing interests related to this research.

## Authors’ contributions

JP developed the concept and research design and drafted the article. JP, TN, and LL collected the data, and all authors contributed substantially to analysis and interpretation of data and critical revision of the manuscript. All authors have read and approved the final manuscript.
